# ThermoPred: AI-Enhanced Quantum Chemistry Data Set and ML Toolkit for Thermochemical Properties of API-Like Compounds and Their Degradants

**DOI:** 10.1021/acs.jcim.5c01320

**Published:** 2025-12-03

**Authors:** Diullio P. Santos, Jefferson R. Dias-Silva, Luiz H. K. Q. Júnior, Heibbe C. B. de Oliveira

**Affiliations:** † Laboratório de Estrutura Eletrônica e Dinâmica Molecular, 67824 Universidade Federal de Goiás Goiânia 74690-900 Goiás, Brasil; ‡ Laboratório de Ciência de Dados e Quimioinformaticar, Universidade Federal de Goiás, Goiânia 74690-900 Goiás, Brasil

## Abstract

In this work, we present an open-access quantum-chemistry database of more than 14,500 API-like molecules and their degradation products, all optimized at the M06-2*X*/6-31G­(d) compound model. The data set delivers a comprehensive suite of thermochemical and quantum descriptors—including Gibbs free energy, enthalpy, electronic energy, vibrational frequencies and Cartesian geometries—tailored for large-scale modeling. Leveraging these data, we trained and validated three machine-learning models (XGBoost, Random Forest and Multi-Layer Perceptron) to enable rapid, accurate prediction of Gibbs free energy and enthalpy. These models are bundled in ThermoPred, an open-source Python package that offers a scalable, computationally efficient alternative to traditional quantum-chemical calculations. All data sets, models and source code are freely available to support reproducibility and foster community-driven development.

## Graphical abstract

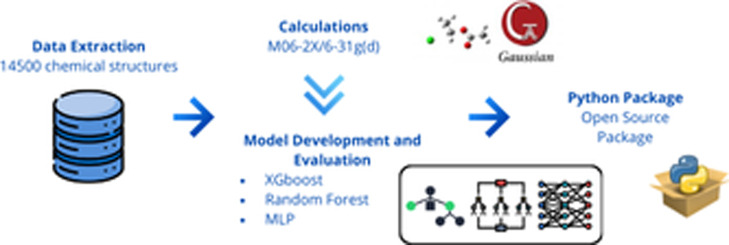

## Introduction

The stability of active? harmaceutical ingredients (APIs) is vital to ensuring the quality, safety and efficacy of drug products over their entire lifecycle.^1^ Regulatory frameworks—such as ANVISA’s RDC 964/2025 and RDC 318/2019, as well as the ICH Q3A/Q3B guidelines—impose strict requirements for the notification, qualification and stability assessment of degradation products.^2,3^ To satisfy these standards, comprehensive stress-testing studies (heat, light, humidity and oxidative conditions) are conducted to identify critical degradation pathways and impurities that could compromise drug safety or therapeutic performance.^4^

Accurate thermodynamic parameters—especially Gibbs free energy and enthalpy—are central to deciphering chemical degradation mechanisms. They enable prediction of reaction spontaneity and feasibility, thereby supporting both regulatory compliance and the rational design of robust, stable formulations. However, high-level quantum-chemical calculations demand substantial computational resources, limiting their routine application in large-scale degradation studies or when screening extensive molecular libraries.^5^ This limitation is particularly evident when evaluating extensive molecular libraries or degradation products, where each molecule requires substantial computational resources for geometry optimization and thermodynamic analysis.

In this context, machine learning (ML) has emerged as a powerful alternative, offering rapid prediction of molecular properties once trained on high-quality quantum-chemistry data sets.^6–9^ ML models can reproduce Gibbs free energies and enthalpies with high fidelity but at a fraction of the computational cost. Yet, publicly available, well-curated data sets tailored for pharmaceutical degradation remain scarce.^10^

To address this gap, based on our expertise in both quantum chemical calculations^11–16^ and machine learning methodologies,^7,17–19^ we introduce an open-access quantum-chemistry data set of more than 14,500 API-like molecules and their degradation products. All structures were fully optimized at the M06-2*X*/6-31G­(d)^20^ level of theory, and their thermodynamic properties—Gibbs free energy, enthalpy, electronic energy, vibrational frequencies, heat capacities, molecular weights and optimized Cartesian coordinates—were calculated and rigorously curated. Generating the complete data set required approximately four months of continuous computation, underscoring its value as a resource for the community.

Leveraging this data set, we developed ThermoPred, an open-source Python package that predicts Gibbs free energy and enthalpy directly from SMILES strings. ThermoPred incorporates pretrained XGBoost, Random Forest and Multi-Layer Perceptron models that emulate quantum-chemical outputs with high accuracy and minimal computational overhead. This tool empowers researchers to perform rapid, large-scale thermodynamic screenings without relying on expensive quantum-chemistry software for every evaluation.

In keeping with our commitment to open science, all data, source code and ML models are freely available under the GNU General Public License v3.0 (GPL-3.0). By fostering transparency and enabling community contributions, ThermoPred and its associated data set offer practical, high-throughput solutions for stability assessment and degradation pathway analysis in computational pharmaceutical research.

## Methodology

### Molecule Selection

The molecular data set was curated from publicly available chemical databases. Active pharmaceutical ingredients (APIs) and their degradation products were primarily selected from the brazilian common names (DCB) list provided by ANVISA and the PubChem database. In addition, a set of molecules similar to APIs and their degradative potentials were collected with the aim of expanding the database. After an initial collection of candidate structures, rigorous preprocessing was applied. Molecules were filtered based on their SMILES and InChIKeys to eliminate duplicates and standardize chemical representations.

The final curated set comprises approximately 14,500 unique API-like molecules. Three-dimensional geometries (XYZ format) were generated from SMILES strings using the RDKit library,^21^ providing accurate starting coordinates for quantum-chemical calculations.

### Density Functional Theory Calculations

Quantum-chemical computations were performed with Gaussian 16 (revision B.01).^22^ Each molecule was fully geometry-optimized at the M06-2*X*/6-31G­(d) level of theory, followed by harmonic frequency analyses to confirm true minima (no imaginary frequencies).

Thermodynamic properties—Gibbs free energy, enthalpy, electronic energy and vibrational frequencies (cm^–1^)—were evaluated at 298.15 K and 1 atm. Optimized Cartesian coordinates were saved in XYZ files for subsequent analysis.

### Molecular Descriptors and Machine Learning Models

Molecular descriptors were generated with the RDKit library^21^ and Morgan fingerprints (radius = 2, 4096 bits) were used as input features, capturing topological characteristics and substructures relevant to thermodynamic-property.

Three machine learning algorithms were implemented and evaluated for the prediction of Gibbs free energy and enthalpy: XGBoost, Random Forest, and Multi-Layer Perceptron (MLP). The XGBoost model^23^ was configured with 1000 decision trees, a maximum depth of 6, and a learning rate of 0.1—parameters chosen to efficiently capture nonlinear relationships in the data set. The Random Forest model^24^ comprised 500 trees with depths optimized automatically, providing strong resistance to overfitting and consistent performance across the high-dimensional chemical descriptor space. The MLP^25^ featured three hidden layers of 512, 256, and 64 neurons, employed ReLU activation functions, and was trained using the Adam optimizer with a learning rate of 0.001, allowing it to flexibly model complex correlations between molecular features and thermodynamic properties.

All models were coded in Python using scikit-learn (v1.3.2)^26^ and XGBoost (v2.0.3) and trained/validated on a high-performance computing cluster, employing the same Morgan-fingerprint inputs to ensure consistent, robust performance comparisons.

### Data Preprocessing and Model Evaluation

All target variables—Gibbs free energy and enthalpy—were first standardized by dividing with the largest value to improve the normality of the variable followed by min max rescaling with a range between 0 and 1. After prediction, values were inverse-transformed to their original scales for direct comparison with reference quantum-chemical results from Gaussian 16.

The values obtained from Gaussian for each molecule correspond to its total molecular energy at the chosen quantum-chemical level–i.e., the electronic energy (including nuclear repulsion) and, when thermochemistry is requested, the thermal corrections that yield H and G. These are absolute quantities and scale primarily with electron count and basis-set size, so they are not directly interpretable on a typical chemical energy scale. Physically meaningful quantities are the variations in energy between states–such as Δ*G* and Δ*H* for reactions, conformer interconversions, or activation barriers–in which the large absolute terms cancel, yielding values on the order of tens to hundreds of kJ·mol^–1^.

Following preprocessing and quality control, the final data set comprised 14,207 molecules with complete thermodynamic profiles. We split this set into training and test subsets using scikit-learn’s train_test_split (random seed = 67857) to guarantee reproducibility: 80% (11,365 compounds) for training and 20% (2842 compounds) held out for external testing.

Model performance was quantified by the coefficient of determination (*R* ^2^),^27^ the predictive squared correlation coefficient (*Q* ^2^),^28^ and the root-mean-square error (RMSE),^29^ offering complementary insights into predictive accuracy and error magnitude. Internal validation employed stratified 10-fold cross-validation on the training set,^30^ ensuring an even distribution of thermodynamic values across folds and mitigating overfitting. Finally, external validation against the independent test set confirmed each model’s generalizability across a chemically diverse collection of API-like molecules and their degradation products.

## Data Records

### Data Set Description

The complete data set is provided as a CSV chemical table that includes all optimized geometries alongside key properties: SMILES, enthalpy, Gibbs free energy, electronic energy, vibrational frequencies, optimized XYZ coordinates, heat capacity, molecular weight (MM) and additional fields (Table 1). This file is freely available in the Supporting Information, and all raw Gaussian log files from the M06-2*X*/6-31G­(d) calculations can be downloaded at http://bit.ly/4jEo7AE.

**Table 1:** Description of Database Fields from Quantum Chemistry Calculations

field	description
electronic energy (SCF Done)	total electronic plus nuclear-repulsion energy at the optimized geometry (hartree)
zero-pointcorrection (ZPE)	zero-point vibrational energy correction (hartree)
thermal correction to internal energy	thermal correction to internal energy U (hartree)
thermal correction to enthalpy	thermal correction to enthalpy *H* (hartree)
thermal correction to Gibbs free energy	thermal correction to Gibbs free energy *G* (hartree)
internal energy	*U* = *E*_SCF_ + thermal correction to internal energy (hartree)
enthalpy	*H* = *E*_SCF_ + thermal correction to enthalpy (hartree)
Gibbs free energy	*G* = *E*_SCF_ + thermal correction to Gibbs free energy (hartree)
heat capacity at constant volume (C_v_)	heat capacity at constant volume (cal·mol^–1^·K^–1^)
entropy	entropy (cal·mol^–1^·K^–1^)
in *Q*_trans_ (translational)	natural logarithm of the translational partition function
in *Q*_rot_ (rotational)	natural logarithm of the rotational partition function
in *Q*_vib_ (vibrational)	natural logarithm of the vibrational partition function
in *Q*_elec_ (Electronic)	Natural logarithm of the electronic partition function
optimized coordinates (XYZ)	cartesian coordinates of the converged geometry (XYZ format)
SMILES	simplified molecular input line entry system string
molecular mass	molecular mass (atomic mass units, amu)

### Exploratory Data Analysis

An initial survey of the data set reveals extensive elemental and functional-group diversity (Table 2). Carbon is the most abundant element (269,734 occurrences), followed by oxygen (67,462) and nitrogen (37,821). Halogens—fluorine, chlorine and bromine—are also well represented, reflecting the typical substitution patterns found in API-like molecules and their degradation products. Functional-group analysis shows a broad distribution across key moieties: ketones (11,035), alcohols (10,029), esters (6,560) and alkenes (5,232), among others, ensuring comprehensive coverage of chemical space critical for developing generalizable thermodynamic models.

**Table 2:** Atomic Elements and Functional Groups Identified in the Dataset

element	count
B	16
Br	13
C	269734
Cl	2520
F	4223
N	37821
O	67462
P	152
S	3629
**functional groups**	**count**
ketones	11035
ethers	1905
alcohols	10029
amines	3043
alkenes	5232
esters	6560
alkynes	243
hydrazines	201

The dimensionality reduction analysis using UMAP, applied to the binary Morgan fingerprints of the molecules in the database, highlights the structural diversity of the “API-like” data set developed in this work. The data set comprises active pharmaceutical ingredients, both real and theoretical degradation products, as well as related impurities, resulting in broad coverage of the chemical space relevant to pharmaceuticals. In the 2D map, each point represents a molecule, colored according to its molecular weight (MolWeight), revealing that most molecules are concentrated in a central region, indicating moderate structural similarity among a large portion of the compounds. However, the presence of peripheral clusters and isolated points suggests the existence of structurally distinct molecules, including likely degradation products and unique impurities, which expand the coverage of the chemical space.

UMAP was chosen over linear techniques such as PCA due to its ability to preserve both the local and global structure of high-dimensional data, which is particularly relevant for molecular fingerprints of binary and sparse nature. The similarity metric adopted was the Jaccard index (equivalent to the Tanimoto coefficient for binary data), as it more appropriately reflects the overlap of “on” bits in the vectors, more accurately capturing chemical similarity between molecules. This combination of technique and metric enabled a realistic representation of similarity relationships within the chemical space, reinforcing the suitability of the database for training machine learning models aimed at predicting physicochemical properties in the pharmaceutical context.

To assess the predictive performance of the developed machine learning models, we calculated the predictive squared correlation coefficient (*Q* ^2^),^28^ the coefficient of determination (*R* ^2^)^27^ and the root-mean-square error (RMSE).^29^ As summarized in Table 3, XGBoost achieved the best performance for Gibbs free energy prediction, with internal validation metrics of *Q* ^2^ = 0.997 ± 0.001, *R* ^2^ = 0.9975 ± 0.0001, and RMSE = 0.009 ± 0.002. The corresponding Pearson correlation coefficient on the external test set (*r*_ext_^2^) was 0.9825, further supporting the strong agreement between predicted and experimental values. For enthalpy prediction, the MLP model outperformed the others in the external validation, reaching *Q*_ext_^2^ = 0.9751, RMSE = 0.0299, and *r*_ext_^2^ = 0.9876, indicating both high predictive accuracy and excellent correlation. The Random Forest model also exhibited consistently high performance across both properties, though slightly below that of XGBoost and MLP in their respective best-performing tasks.

**Table 3:** Predictive Performance of the XGBoost, Random Forest, and MLP Models for Gibbs Free Energy and Enthalpy^a^

external validation (Gibbs free energy)				
	*Q*_ext_^2^	*R*_ext_^2^	**RMSE**	*r*_ext_^2^
**XGBoost**	0.9653	0.9975	0.0353	0.9825
**random forest**	0.9361	0.9880	0.0479	0.9685
**MLP**	0.9773	0.9911	0.0285	0.9889
**internal validation (Gibbs free energy)**				
**XGBoost**	0.997 (±0.001)	0.9975 (±0.0001)	0.009 (±0.002)	0.9988 (±0.0005)
**random forest**	0.988 (±0.001)	0.9879 (±0.0001)	0.021 (±0.001)	0.9944 (±0.0006)
**MLP**	0.9911 (±0.0008)	0.99106 (±0.00009)	0.00032 (±0.00003)	0.9958 (±0.0005)
**external validation (enthalpy)**				
**XGBoost**	0.9433	0.9663	0.0451	0.9716
**random forest**	0.9363	0.9879	0.0478	0.9687
**MLP**	0.9751	0.9896	0.0299	0.9876
**internal validation (enthalpy)**				
**XGBoost**	0.966 (±0.002)	0.9663 (±0.0002)	0.0346 (±0.0008)	0.983 (±0.001)
**random forest**	0.988 (±0.001)	0.9879 (±0.0001)	0.021 (±0.001)	0.9944 (±0.0006)
**MLP**	0.990 (±0.001)	0.9896 (±0.0001)	0.00037 (±0.00004)	0.9948 (±0.0006)

a Internal validation results are expressed as mean ± standard deviation over 10-fold stratified cross-validation. External validation metrics were computed on an independent test set.

Both internal and external validation procedures—including stratified 10-fold cross-validation on the training set^30^ and hold-out testing on the independent set—were conducted to assess model robustness and generalization capability. The close agreement between internal and external metrics indicates minimal overfitting, confirming that the models reliably predict thermodynamic properties across a chemically diverse collection of API-like molecules and their degradation products. These findings underscore the applicability of our approach to high-throughput screening and predictive modeling in pharmaceutical research (see Figure 1).

**Figure 1:**
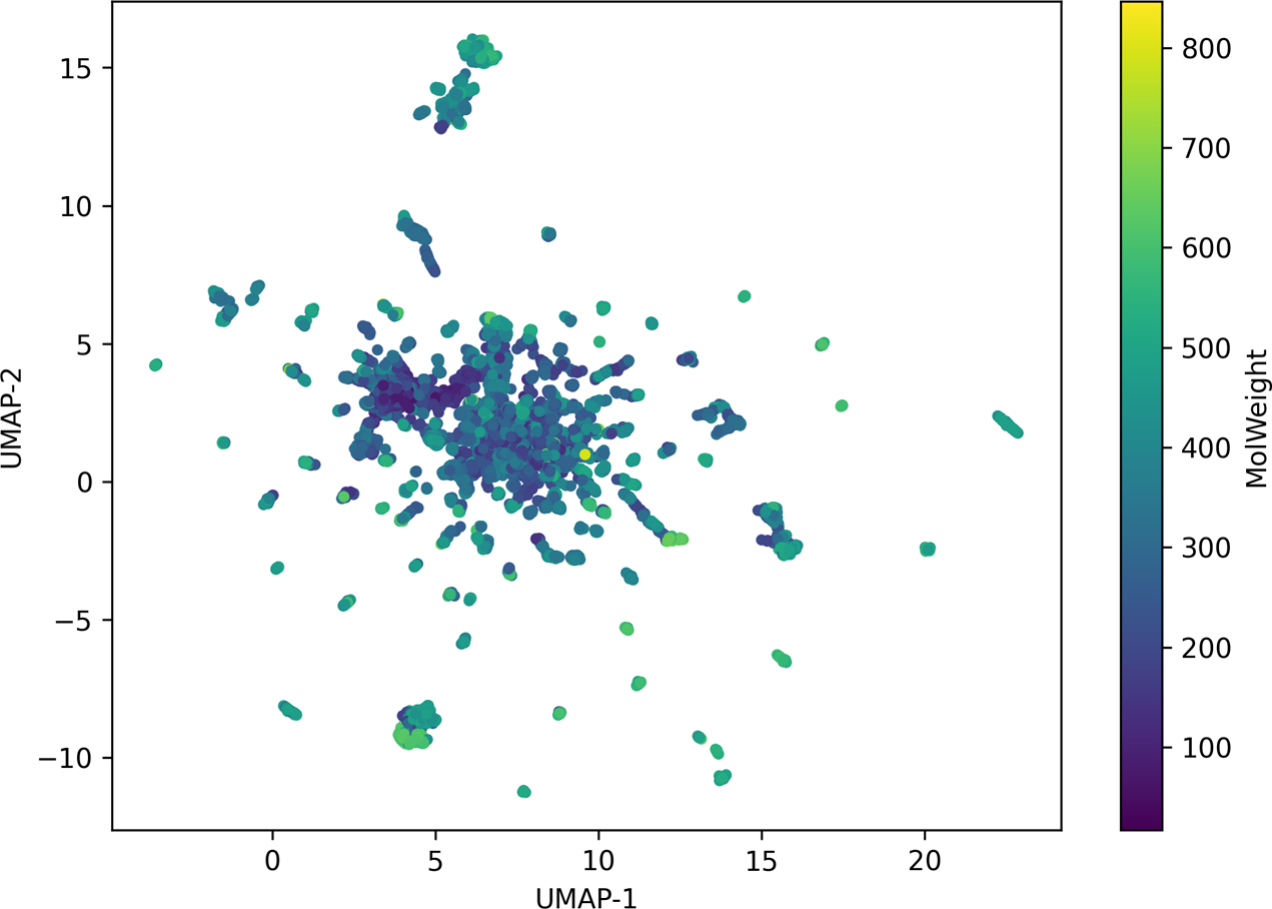
Two-dimensional map obtained by UMAP from the binary Morgan fingerprints of the API-like data set. Each point represents a molecule, colored according to its molecular weight (MolWeight). The data set includes active pharmaceutical ingredients, real and theoretical degradation products, and related impurities.

Figure 2 presents parity plots comparing predicted versus reference values for Gibbs free energy and enthalpy across the MLP, Random Forest and XGBoost models. All values were normalized between 0 and 1 to facilitate direct comparison. The red diagonal line represents the ideal Pearson’s correlation (*r* ^2^ = 1),^31^ where predicted and experimental values coincide. Points clustering close to this line denote high predictive accuracy, while deviations reflect over- or under-predictions.

**Figure 2:**
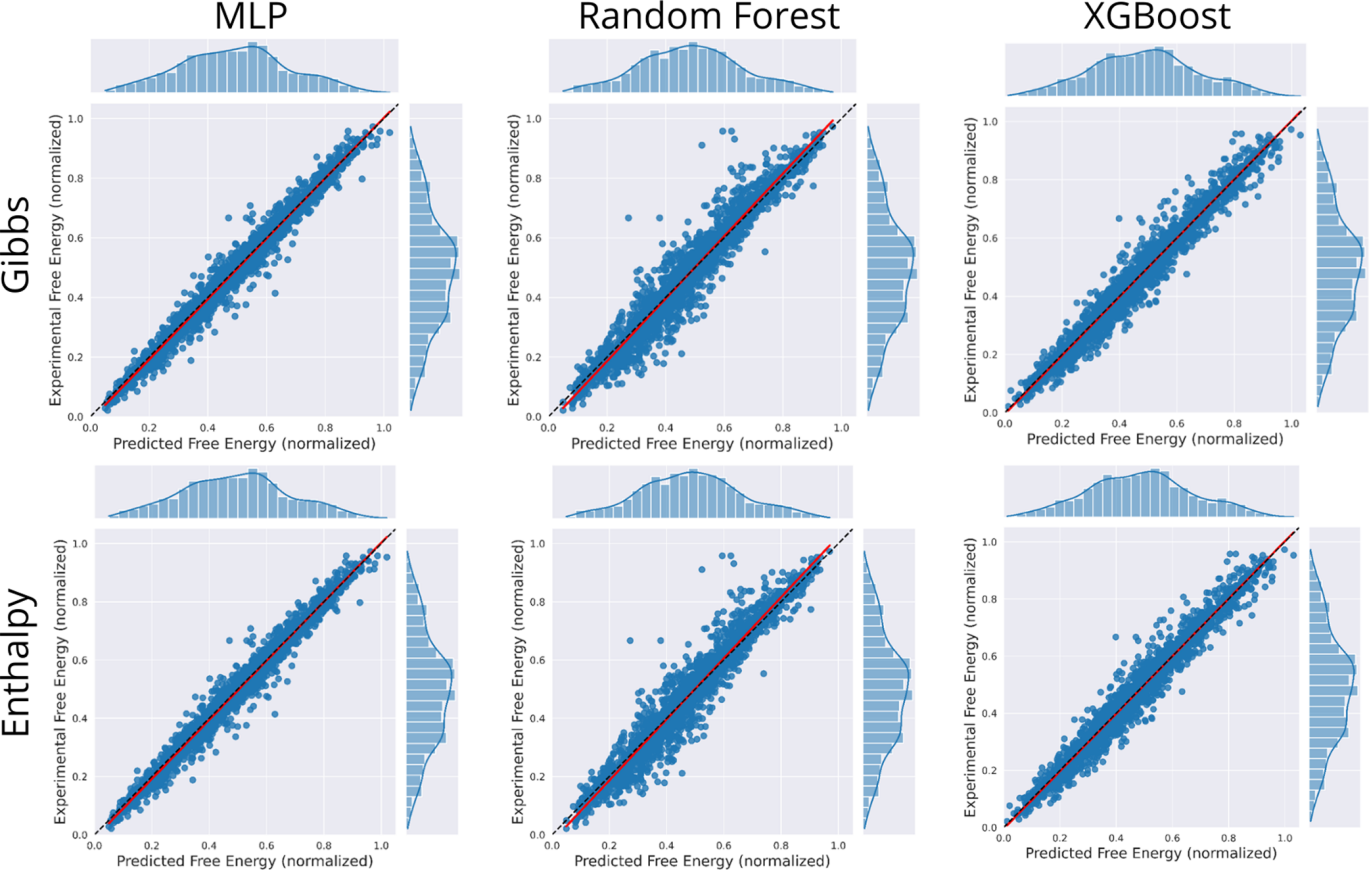
Parity plots comparing predicted versus experimental values (normalized) for Gibbs free energy (top row) and enthalpy (bottom row), using three machine learning models: Multi-Layer Perceptron (MLP), Random Forest, and XGBoost. The red diagonal line represents the ideal correlation (*r* ^2^ = 1), where predicted and experimental values are equal. Data points closely aligned with this line indicate accurate predictions, while deviations reflect under- or overpredictions by the models. Histograms above and beside each scatter plot illustrate the distribution of predicted and experimental values, respectively.

For Gibbs free energy predictions (top row in Figure 2), XGBoost achieves the closest alignment with the ideal diagonal, exhibiting minimal scatter and underscoring its superior predictive accuracy. The MLP delivers comparably strong performance but shows slightly greater variance around the reference line, while Random Forest displays a broader spread, particularly at intermediate values, indicating less consistent predictions.

In the case of enthalpy (bottom row in Figure 2), MLP outperforms both tree-based models, with points tightly clustered along the diagonal and minimal deviation across the normalized range. XGBoost follows closely, maintaining good concordance between predicted and reference values, whereas Random Forest again presents larger dispersion, especially at the extremes.

Taken together, these observations—supported by stratified 10-fold cross-validation and external validation metrics (Table 3)—demonstrate the robustness and generalizability of our models. In particular, XGBoost for Gibbs free energy and MLP for enthalpy prediction show strong potential for accurate, high-throughput thermodynamic screening of API-like molecules and their degradation products.

To assess the applicability domain (AD) of the models, we calculated the Tanimoto similarity between each molecule in the external test set and those in the training set using Morgan fingerprints (radius = 2, nBits = 4096). As shown in Figure 3, the majority of test compounds exhibit a similarity above 0.6, with a distribution peak between 0.7 and 0.85, suggesting that most predictions are made within a well-represented chemical space. This supports the reliability of the external validation results, as the models are not extrapolating to structurally dissimilar regions. Only a small number of molecules showed similarity below 0.4, indicating limited risk of out-of-domain predictions.

**Figure 3:**
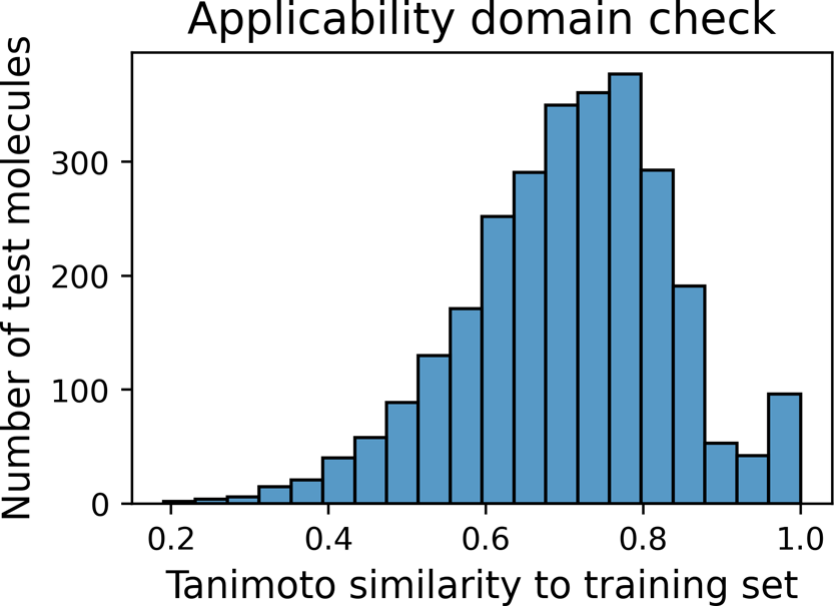
Distribution of the Tanimoto similarity values between molecules in the test set and those in the training set, used to assess the applicability domain of the predictive models.

The Bemis-Murcko scaffold analysis^32^ (Figure 4), which isolates the core structures (frameworks) of molecules by removing peripheral substituents, revealed the predominance of aromatic and heteroaromatic systems in the data set, reflecting the “API-like” nature of the collection. The simple benzene ring was the most frequent core, appearing in 1042 molecules, followed by fused or connected bicyclic structures such as biphenyl (129 occurrences) and tetrahydro-β-carboline (108 occurrences). Several heterocycles containing oxygen, nitrogen, and sulfur—such as morpholine, piperazine, and thiazoles—also ranked among the most common, indicating the significant presence of pharmacophoric fragments typical of drugs and related impurities. The diversity of cores, ranging from simple aromatic systems to condensed polycyclic structures and heterocycles of varying sizes, reinforces the broad coverage of the chemical space relevant to pharmaceutical products. These findings complement the dimensionality reduction (UMAP) analysis and the applicability domain study, confirming that the data set combines structural motifs widely represented in marketed drugs with rarer cores, possibly associated with specific degradation products and impurities. The entire script created to perform validations and exploratory analyses can be accessed through the link: https://github.com/jeffrichardchemistry/thermopred/blob/main/validation/Validation_Scripts.ipynb.

**Figure 4:**
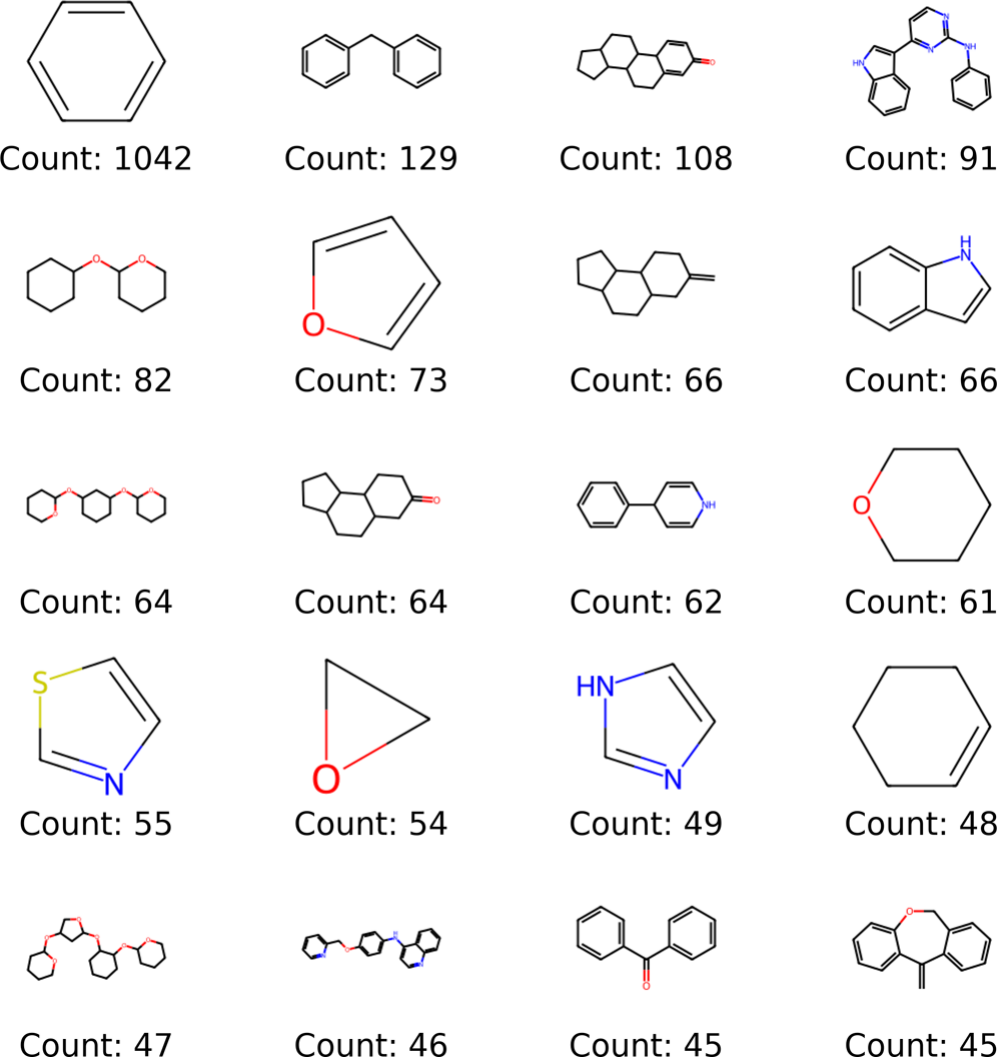
Top 20 most frequent Bemis-Murcko scaffolds identified in the data set, highlighting the prevalence of aromatic and heteroaromatic cores typical of API-like molecules.

### Implementation

In this work, we provide an open-source Python package, Thermopred, along with a comprehensive data set containing thermochemical and quantum mechanical data for approximately 14,500 API-like molecules and their degradation products. The goal is to enable rapid and reliable prediction of Gibbs free energy and enthalpy, simulating the outputs typically obtained through quantum chemical calculations performed with software such as Gaussian. All data and source code are openly available at https://github.com/jeffrichardchemistry/thermopred.

### Python Package Overview

The ThermoPred package has been designed to provide an intuitive and user-friendly interface for predicting thermodynamic properties from molecular SMILES strings. It is implemented in Python and offers pretrained machine learning models, including XGBoost, random forest, and multi-layer perceptron (MLP), all optimized for accuracy and computational efficiency. The repository is structured as shown in Figure 5.

**Figure 5:**
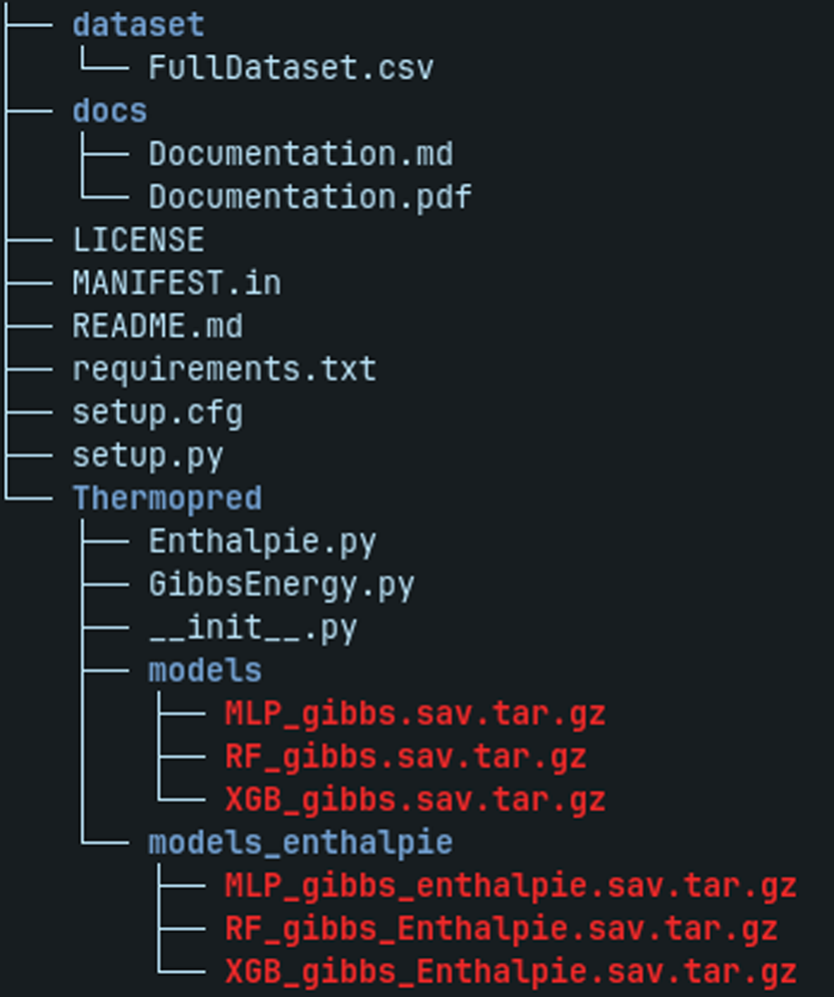
Directory structure of the Thermopred project. The data set folder contains the main input data file (FullData set.csv). The docs folder includes the project documentation in both Markdown and PDF formats. Files such as LICENSE, MANIFEST.in, README.md, requirements.txt, setup.cfg, and setup.py are located in the project root directory and provide installation configurations and dependency management. The Thermopred directory contains the Python modules responsible for calculating thermodynamic properties, including Enthalpie.py and GibbsEnergy.py, as well as two subdirectories (models and models_enthalpie) that store the machine learning models trained for predicting Gibbs free energy and enthalpy, respectively.

### Functionality and Usage

ThermoPred provides a streamlined Python interface for predicting thermodynamic properties, specifically Gibbs free energy and enthalpy, from molecular SMILES strings. The package includes pretrained machine learning models—XGBoost, Random Forest, and MLP—which were trained and validated to ensure accurate and reliable predictions. These models are designed to simulate the outputs typically generated by quantum chemistry software, such as Gaussian 16, and return results in the same units, facilitating seamless integration into computational workflows.

The package can be easily installed and executed within Python environments. To predict Gibbs free energy and enthalpy, users only need to import the corresponding modules and provide a SMILES string as input. Figure 6 illustrates this workflow, showing prediction outputs for a representative API-like molecule.

**Figure 6:**
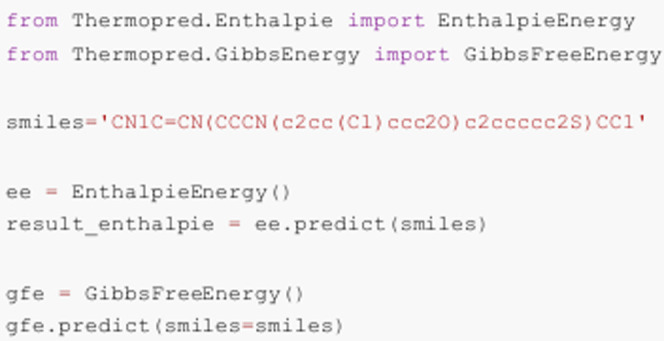
Example Python code demonstrating the use of the ThermoPred package for predicting thermodynamic properties. The script shows how to predict Gibbs free energy and enthalpy from a SMILES string using the pretrained machine learning models available in ThermoPred.

As an open-source project, ThermoPred welcomes contributions—such as incorporating additional descriptor types, retraining on custom data sets or improving the interface—and is designed as a flexible platform for high-throughput prediction of thermodynamic properties in API-like compounds and their degradation products.

## Conclusions

In this work, we present an open-access, meticulously curated quantum-chemistry database of over 14,500 API-like molecules and their degradation products. Each structure was optimized with Gaussian 16 at the M06-2*X*/6-31G­(d) level, yielding a rich set of thermochemical and electronic properties—Gibbs free energy, enthalpy, electronic energy, vibrational frequencies—and Cartesian geometries. Generating this resource required approximately 4 months of continuous computation, underscoring the substantial investment in processing time and energy and delivering a valuable tool for the scientific community.

Beyond serving as a robust foundation for pharmaceutical stability and degradation studies, our database provides an essential benchmark for developing and validating predictive models. To leverage these data, we developed ThermoPred, an open-source Python package that embeds three machine-learning models—XGBoost, Random Forest and Multi-Layer Perceptron—trained on our curated data set. ThermoPred delivers accurate, rapid predictions of Gibbs free energy and enthalpy, dramatically reducing the need for costly quantum-chemical calculations.

By combining a large-scale, high-quality data set with validated machine-learning tools, this work enables high-throughput screening, stability assessments and mechanistic investigations in pharmaceutical research. Making both the database and ThermoPred freely available promotes transparency, reproducibility and collaborative development. We anticipate that this platform will accelerate thermodynamic-property prediction for drug-like molecules and drive future advances in computational pharmaceutical chemistry.

## Supplementary Material





## Data Availability

The ThermoPred package and models are available for Windows, Linux, and macOS, and can be downloaded from the GitHub repository (https://github.com/jeffrichardchemistry/thermopred). The package is also distributed via PyPI for direct installation. In addition, an interactive web interface is available for testing through Streamlit. The license for the Python package and models is GPL-3.0. All molecular structures used for each modeled data set are provided in the Supporting Information. The contents of all Gaussian output files (logs) can be downloaded via the following link: https://huggingface.co/datasets/diullio/ThermoPredLogs.

## Notes

Complete data set used for creating and validating ML models and also contains data extracted from all Gaussian output files (PDF)
The data set folder contains the main input data file (FullData set) (CSV)
