# Supplementary material for: ThermoPred: AI-Enhanced Quantum Chemistry Data Set and ML Toolkit for Thermochemical Properties of API-Like Compounds and Their Degradants
Source: J Chem Inf Model. 2025 Dec 3;65(24):13004–11. doi: 10.1021/acs.jcim.5c01320 (PMC12728917; doi:10.1021/acs.jcim.5c01320)
Supplement: Supplementary file 1 [file ci5c01320_si_001.pdf]

Supporting Information.

ThermoPred: AI-Enhanced Quantum Chemistry Dataset and ML Toolkit for Thermochemical Properties of API-Like Compounds and Their Degradants

Diullio P. Santos[†], Jefferson R. Dias-Silva[‡], Luiz H. K. Q. Júnior[‡], and Heibbe C. B. de Oliveira[†]

[†]Laboratório de Estrutura Eletrônica e Dinâmica Molecular, Universidade Federal de Goiás, Goiânia 74690-900, GO, Brasil.

[‡]Laboratório de Ciência de dados e Quimioinformatica, Universidade Federal de Goiás, Goiânia 74690-900, GO, Brasil.

Description of the Database

A computational database comprising over 14,500 API-like molecular structures was generated in this work. For each molecule, quantum chemical calculations were performed at the M06-2X/6-31G(d) level of calculation to obtain optimized geometries and thermochemical parameters. The resulting data include total electronic energies, thermal and zero-point corrections, and statistical thermodynamics quantities, all extracted directly from the *Gaussian* output files. Each entry in the dataset represents a single optimized molecule and includes the following fields:

- **Id** – Unique identifier for each molecule in the database.
- **SCF_Energy** – Self-consistent field (SCF) total electronic energy obtained from the final geometry optimization step (in Hartree).
- **Gibbs_Energy_SCF+G_corr** – Gibbs free energy including the electronic SCF energy and thermal correction to Gibbs energy as reported by *Gaussian* ("Sum of electronic and thermal Free Energies").
- **Enthalpie_SCF+H_Corr** – Enthalpy including the electronic SCF energy and thermal correction to enthalpy ("Sum of electronic and thermal Enthalpies").
- **Energy_thermal_SCF+U_Corr** – Internal energy including the electronic SCF energy and thermal correction ("Sum of electronic and thermal Energies").
- **Energy_ZeroPoint_SCF+ZPE_Corr** – SCF energy corrected by the zero-point vibrational energy (ZPE).

- **Gibbs_Corr / Enthalpie_Corr / Energy_thermal(U)_Corr / ZPE_corr** – Corresponding thermal and zero-point corrections extracted individually from the thermochemistry section of *Gaussian*.

- **Rotational_LnQ / Translational_LnQ / Electronic_LnQ / VibrationalBot_LnQ / VibrationalV0_LnQ** – Natural logarithm of the rotational, translational, electronic, and vibrational partition functions, as reported in the *Gaussian* frequency analysis output.

- **InternalEnergy_KCal/Mol, HeatCapacity_Cal/Mol-Kelvin, Entropy_Cal/Mol-Kelvin** – Thermodynamic quantities converted to common energy units ($kcal \cdot mol^{-1}$ and $cal \cdot mol^{-1} \cdot K^{-1}$).

- **MolOPT_XYZ** – Optimized molecular geometry in XYZ format, corresponding to the minimum-energy structure after frequency verification (no imaginary frequencies).

- **Smiles** – Canonical SMILES representation of the optimized structure, used to link quantum descriptors with cheminformatics tools.

- **Mass** – Molecular mass (in atomic mass units) calculated from the optimized molecular formula.

This dataset integrates quantum and thermochemical information into a single structured resource suitable for machine learning, molecular similarity, and property prediction studies. It provides a comprehensive foundation for analyzing the relationship between molecular structure, quantum energy components, and thermodynamic

stability in drug-like chemical space. The file can be downloaded at: https://github.com/jeffrichardchemistry/thermopred/blob/main/dataset/FullDataset.csv
